# CONSORT to community: translation of an RCT to a large-scale community intervention and learnings from evaluation of the upscaled program

**DOI:** 10.1186/s12889-017-4907-2

**Published:** 2017-11-29

**Authors:** Carly Jane Moores, Jacqueline Miller, Rebecca Anne Perry, Lily Lai Hang Chan, Lynne Allison Daniels, Helen Anna Vidgen, Anthea Margaret Magarey

**Affiliations:** 1 Nutrition and Dietetics, College of Nursing and Health Sciences, Flinders University, Adelaide, South Australia Australia; 2 School of Exercise and Nutrition Sciences, Faculty of Health, Queensland University of Technology, Brisbane, QLD Australia

**Keywords:** Child obesity, Effectiveness, Evaluation, Family, Implementation, Intervention, Lifestyle, Parenting, Translation

## Abstract

**Background:**

Translation encompasses the continuum from clinical efficacy to widespread adoption within the healthcare service and ultimately routine clinical practice. The Parenting, Eating and Activity for Child Health (PEACH™) program has previously demonstrated clinical effectiveness in the management of child obesity, and has been recently implemented as a large-scale community intervention in Queensland, Australia. This paper aims to describe the translation of the evaluation framework from a randomised controlled trial (RCT) to large-scale community intervention (PEACH™ QLD). Tensions between RCT paradigm and implementation research will be discussed along with lived evaluation challenges, responses to overcome these, and key learnings for future evaluation conducted at scale.

**Methods:**

The translation of evaluation from PEACH™ RCT to the large-scale community intervention PEACH™ QLD is described. While the CONSORT Statement was used to report findings from two previous RCTs, the REAIM framework was more suitable for the evaluation of upscaled delivery of the PEACH™ program. Evaluation of PEACH™ QLD was undertaken during the project delivery period from 2013 to 2016.

**Results:**

Experiential learnings from conducting the evaluation of PEACH™ QLD to the described evaluation framework are presented for the purposes of informing the future evaluation of upscaled programs. Evaluation changes in response to real-time changes in the delivery of the PEACH™ QLD Project were necessary at stages during the project term. Key evaluation challenges encountered included the collection of complete evaluation data from a diverse and geographically dispersed workforce and the systematic collection of process evaluation data in real time to support program changes during the project.

**Conclusions:**

Evaluation of large-scale community interventions in the real world is challenging and divergent from RCTs which are rigourously evaluated within a more tightly-controlled clinical research setting. Constructs explored in an RCT are inadequate in describing the enablers and barriers of upscaled community program implementation. Methods for data collection, analysis and reporting also require consideration. We present a number of experiential reflections and suggestions for the successful evaluation of future upscaled community programs which are scarcely reported in the literature.

**Trials registration:**

PEACH™ QLD was retrospectively registered with the Australian New Zealand Clinical Trials Registry on 28 February 2017 (ACTRN12617000315314).

## Background

Implementation science is an emerging area of research that studies the translation of evidenced-based interventions into routine practice and is particularly applicable to healthy lifestyle public health initiatives. The ideal translation continuum begins with an evidence-based randomised controlled trial (RCT) to establish efficacy, followed by community trials to determine clinical effectiveness (i.e. does the intervention work in the real world setting) and finally, adoption and routine delivery by health care delivery systems [1]. The translation of an intervention into the community setting is an iterative process requiring adaptation of the intervention itself, adoption of the program by the community, and implementation often under quite different conditions to the original RCT. Similarly, the evaluation of the intervention evolves with the move from RCT to a program delivered by health providers rather than researchers. Evaluation of an RCT aims to demonstrate efficacy, with little emphasis on process evaluation which probes how the program is received, understood and utilised by participants. Evaluation priorities of community programs include program reach, uptake, fidelity and sustainability to ascertain the feasibility of incorporating the program into routine health service delivery which account for broader contextual social, political and economic factors associated with successful program implementation [2].

While there is a growing body of literature on changes to the implementation of healthy eating programs across the translation continuum, there is little dissemination of the corresponding changes in evaluation, or reflections on evaluation challenges once upscaled. The aim of this paper is to describe the translation of the evaluation framework from a randomised controlled trial (RCT) to a large-scale community intervention using the PEACH™ Program as a case study. This paper also aims to provide the authors’ experiential reflections on their lived tension between RCT and implementation research paradigms, and provide examples along with key learnings which are beneficial for future evaluation of upscaled programs to assist the implementation of programs along the translation pathway.

## Methods

### The PEACH™ program

Child obesity is a global public health crisis which requires the implementation of available and effective programs [3]. The recent World Health Organization (WHO) Report of the Commission on Ending Childhood Obesity recommends the provision of family-based, multicomponent, lifestyle weight-management services for children and young people who are obese [4]. The Parenting, Eating and Activity for Child Health (PEACH™) Program [5–7] is a parent-led, family-focussed program for families of overweight children which aligns with the aforementioned WHO recommendation. PEACH™ is a 6-month parent-led lifestyle program consisting of 10× 90-min group sessions for parents which are facilitated by a qualified health professional (e.g. dietitian, nutritionist) who had received training in the program. Between sessions 9 and 10 there are three one-on-one phone calls with facilitators which provide individualised support and encouragement to maintain and implement additional healthy lifestyle changes. Children and any siblings attend concurrent group sessions which involved facilitated group-based physical activity and games. The PEACH™ Program aims to support parents in managing their children’s weight by taking a whole-of-family approach and providing information, confidence and skills regarding family nutrition and physical activity, parenting, and problem solving.

**Fig. 1:**
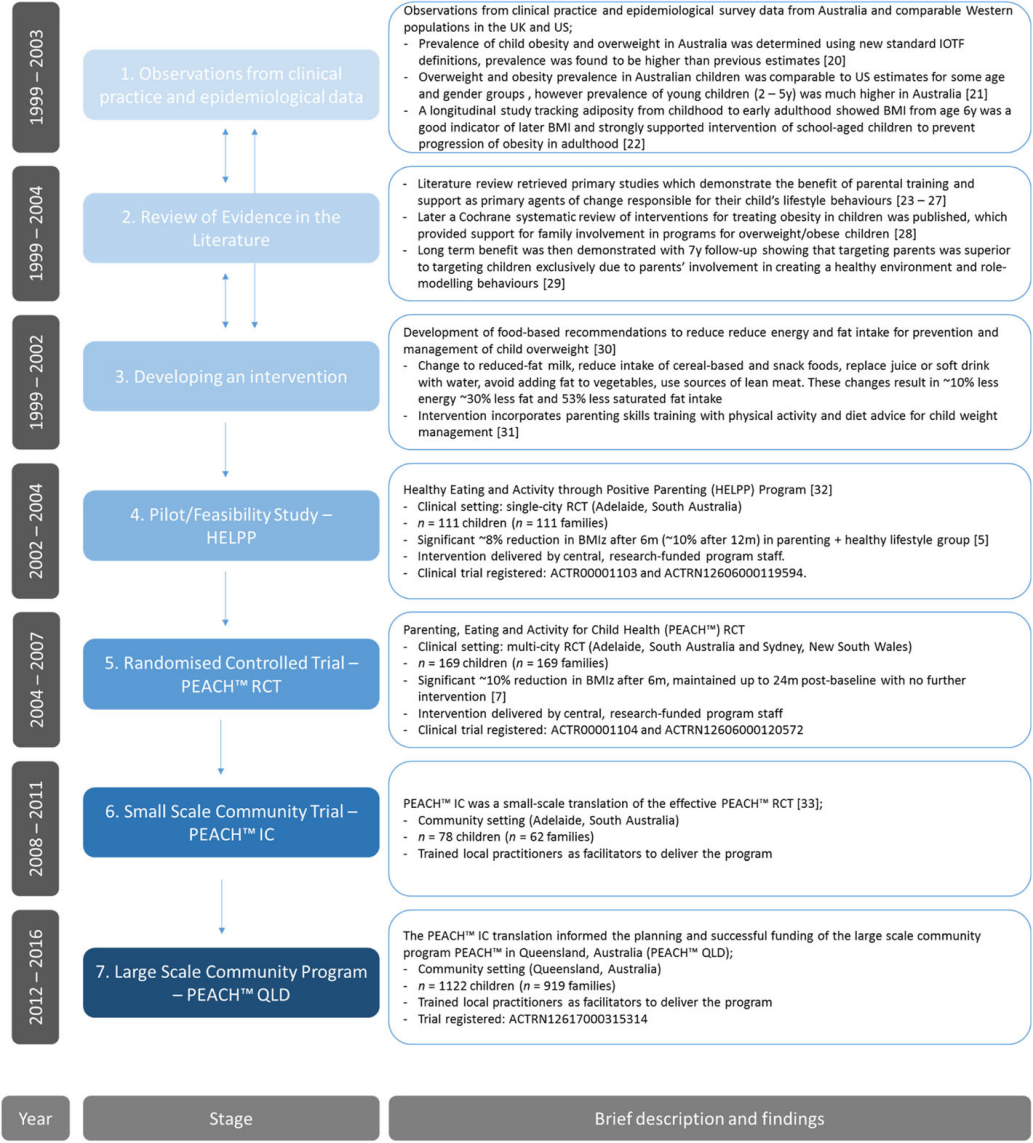
Translational pathway of the PEACH™ program (with approximate timeline). *Abbreviations; HELPP, Healthy Eating and Activity through Positive Parenting; m, month; PEACH™, Parenting, Eating and Activity for Child Health; y, year*. Cited literature [5, 7, 20–33]

The translational pathway of the PEACH™ Program to-date is depicted in Fig. 1. Briefly, the program has progressed from conception across the translation continuum over approximately 17 years. We observed the largest reduction in BMI z-score in the Healthy Eating and Activity through Positive Parenting (HELPP) and PEACH™ RCTs (8% and 10% reduction at 6 m, respectively) compared to the community trial PEACH™ IC and the state-wide population health program PEACH™ QLD (approximately 5–6% reduction), which is a finding in line with previous reports that efficacy trials can over-estimate the effect of an intervention [8].

### Translation of evaluation from PEACH™ RCT to PEACH™ QLD

An overview of the evaluation in each of the three selected PEACH™ iterations to-date is shown (Table 1). PEACH™ RCT was registered as a clinical trial (ACTR00001104 and ACTRN12606000120572) and reported against the (CONSORT) Statement [9, 10]. PEACH™ IC and PEACH™ QLD were community trials reported against the REAIM framework. PEACH™ QLD was retrospectively registered with the Australian New Zealand Clinical Trials Registry on 28 February 2017 (ACTRN12617000315314).

**Table 1:** Overview and evaluation of three PEACH™ iterations to-date

Stage of translation:	Stage 1: Randomised-Controlled Trial (CONSORT)	Stage 2: Small-scale community trial (COMMUNITY I)	Stage 3: Large-scale community intervention (COMMUNITY II)
	PEACH™ RCT [7]	PEACH™ IC [33]	PEACH™ QLD
*n* participants	*n* = 169 families; *n* = 169 children	*n* = 62 families; *n* = 78 children	*n* = 919 families; *n* = 1122 children
*n* groups	*n* = 6	*n* = 8	*n* = 105
*n* facilitators	*n* = 3 delivered sessions	*n* = 12 delivered sessions; *n* = 54 trained	*n* = 52 delivered sessions; *n* = 80 trained
Program overview and setting	▪ Single-blinded RCT with 2 intervention groups (1) 12× parenting (P) and healthy lifestyle (HL) group sessions OR (2) 8× HL group sessions 90- to 120-min group sessions, both with 4× one-to-one phone calls, delivered over 6 months with tapered frequency (weekly, fortnightly, then monthly) ▪ Multi-site (Sydney and Adelaide)	▪ 10× 90-min fortnightly face-to-face group HL sessions incorporating P skills with 3× one-to-one phone calls over 6 months ▪ South Australia	▪ 10× 90-min face-to-face group HL sessions incorporating P skills, with 3 one-to-one phone calls over 6 months ▪ Sessions 1–9 initially held fortnightly, changed to weekly during program delivery ▪ Queensland
Evaluation			
Trial registration	✓ ACTR00001104; ACTRN12606000120572	✗	✓ ACTRN12617000315314
Program delivery (logistics)	✓	✓	✓
Demographics	✓ family, parent and child	✓ family, parent and child	✓ family, parent and child
Anthropometry	✓ parent and child	✓ child	✓ child
Child diet	✓ parent-reported	✓ parent-reported	✓ parent-reported
Child activity	✓ parent-reported	✓ parent-reported	✓ parent-reported
Child quality of life	✓ parent- and child-reported	✗	✓ child-reported
Parenting	✓ parent-reported	✗	✓ parent-reported
Program satisfaction	✓ parent-reported	✓ parent-reported	✓ parent- and child-reported
Child body image	✓ child-reported	✓ child-reported	✗
Follow-up	✓ up to 5 years	✓ only to 6 months	✓ only to 6 months
Program fidelity	✓ independently assessed from audio recordings of sessions	✓ informal only	✓ facilitator-reported
Facilitator training/delivery	✓	✓ pre- and post-training, post-delivery	✓ pre- and post-training, post-delivery
Clinical biochemistry	✓ child	✗	✗
Follow-up parent interviews	✓ 12 months	✗	✗
Service-level evaluation	✗	✓	✓
System-level evaluation	✗	✗	✓

*HL* healthy lifestyle*, P* parenting

The evaluation framework and approach was carried forward from the RCT to the community setting with relatively few changes as the program progressed from demonstrating efficacy in an RCT to determining effectiveness in small- and large-scale community trials.

### PEACH ™ QLD

In 2012, the Queensland Department of Health awarded a tender to Queensland University of Technology (QUT) to deliver the PEACH™ family-focussed, parent-led, child weight management program to 1400 Queensland children in order to: 1) increase the capacity of the families who participate to adopt healthy lifestyles related to healthy eating and physical activity; and 2) promote healthy weight and weight management through sustainable behaviour change. Rather than a research project, PEACH™ QLD was first and foremost a community service delivery project. The team at QUT were responsible for the implementation and delivery of the PEACH™ QLD Project (Project Implementation Team). Flinders University (Adelaide, South Australia) was subcontracted by QUT as external evaluators of the program (Evaluation Team) with the funder prescribing the required effectiveness outcomes in the tender. The evaluation of PEACH™ QLD was funded by the service delivery tender awarded to QUT. The evaluation framework, including the research questions and tools proposed by the QUT and Flinders University teams, was subject to approval by the funder, the Queensland Department of Health. Amendments required by the funder were limited to the use of standardised demographic questions for comparability with population monitoring and surveillance conducted by the Department. No restrictions were placed on the reporting of data, however communication of results during the funding period was also subject to approval. As evaluators of PEACH™ QLD, Flinders University were responsible to the Project Implementation Team at QUT. Ultimately QUT were responsible for the delivery of the project to Queensland Health. Queensland Health received interim reports on program outcomes approximately 6-monthly. Along with project updates, consultation with facilitators and feedback from families, these regular evaluation reports both precipitated and informed changes to program implementation and evaluation during the project lifecycle which were approved by the funder.

PEACH™ originated at Flinders University and so the evaluators had intimate knowledge of the program and had conducted the first RCT and small scale community trial. Comprehensive evaluation on program outcomes, impact and process indicators, as well as assessment of adherence to program protocol and implementation were tailored to fit the upscaled program from the RCT evaluation framework. In contrast to the RCT, parent-completed questionnaires were administered on-line using Survey Monkey. This modification was designed to enable immediate data access to the evaluators and to reduce administrative time and cost associated with printing, distributing, collecting and returning hard copies, as well as data entry. Tablet computers were provided to parent facilitators to allow parents to complete questionnaires at the first session if not done prior.

The evaluation of the PEACH™ QLD Project was adapted to the REAIM framework components of Reach (an individual-level measure of patient participation and representativeness); Effectiveness (the program’s success rate at an individual level); Adoption (program acceptance/uptake at the organisational level); Implementation (fidelity of the program to the original RCT intervention, measured at the organisational level); and Maintenance (long term effects at the individual and organisational level) [11]. Table 2 briefly defines REAIM framework components and within each of these dimensions details the outcome, impact and process evaluation collected during the PEACH™ QLD Project. This paper does not report the outcomes of these measures but instead provides experiential learnings and reflections in conducting the evaluation described in the framework in a real-world service delivery project.

**Table 2:** Evaluation data collected for the PEACH™ QLD Project against the RE-AIM framework dimensions

RE-AIM dimension and definition		Level (source) of data	Data collected (O/I/P)^a^	Tool used/items generated	Further detail and references
REACH	*Proportion of the target population that participated in the intervention*	Individual (Family)	Number of families enrolled (P)	Purpose-developed database	Recruitment and enrolment databases developed, unique nine-digit ID allocated at enrolment
			Family demographics (P)	Questionnaire	Family demographics included family composition, parent education, ethnic background and income level. It is adapted from a previously used data collection form [34].
EFFICACY/EFFECTIVENESS	*Success rate if implemented as in guidelines; defined as positive outcomes minus negative outcomes*	Individual (Facilitator)	Changes in knowledge, skills and confidence (I)	Purpose-developed questionnaire	Self-rated on a Likert scale for the practice areas of family-focussed weight management, lifestyle support, behaviour modification
			Satisfaction with program training and resources (P)	Purpose-developed questionnaire	Parent facilitator satisfaction with program training workshop and program resources was collected pre- and post-training, and post delivery
		Individual (Child)	Child anthropometric measures (O)	Standardized measures for weight, height, waist circumference	WHO2007 [35], US-CDC2000 [36] and UK1990 [37] BMI z-scores and UK1990 WC z-scores; children categorised using IOTF thresholds [38, 39]
			Parent-reported child diet (O)	Children’s Dietary Questionnaire (CDQ) scores for 1) Fruits & vegetables; 2) Sweetened beverages; 3) Fat from dairy products; 4) Discretionary foods; and 5) Food behaviours	Forty-item semi-quantitative dietary questionnaire validated to assess diet quality and food behaviours of school-aged children against the Australian Dietary Guidelines [40, 41]
				Core food group serves for: 1) Fruits; 2) Vegetables; 3) Grains; 4) Meats and alternatives; and 5) Dairy and alternatives	Ten-item, parent completed questionnaire to assess intake of the five core food groups of Australian Guide to Healthy Eating (AGHE) validated in a sample of 45 [31].
			Parent-reported child physical activity and sedentary behaviours (O)	Children’s Leisure Activities Study Survey (CLASS)	Assessed using the Children’s Leisure Activities Study Survey (CLASS) questionnaire [42], modified to focus on active pastimes and screen-time only. Provides a quantitative estimate of children’s time spent in moderate, vigorous and total physical activity, and in screen-based sedentary activities per day. The parent-completed version was used as it is equal in validity and reliability to the child-completed questionnaire [42], and allowed consistency in survey administration of diet and PA outcomes.
			Child-reported health-related quality of life (I)	Child Health Utility 9D (CHU9D)	9 item self-completed paediatric generic preference-based measure of health-related quality of life [43]. It gives a utility value for each health state described from which quality adjusted life years (QALYs) can be calculated. Validated in 7 to 17 year olds [44].
			Child program satisfaction (P)	Purpose-developed group activity and questionnaire	Children’s views of their group sessions were captured via a brief questionnaire and informal group discussion in the last session.
		Individual (Family)	Parenting self-efficacy (I)	Parenting self-efficacy	Four-item questionnaire from the Longitudinal Study of Australian Children [45].
			Parent barriers, confidence and health beliefs (I)	Purpose-developed questionnaire	Five-item purpose-developed tool to assess parent beliefs about their child’s health, and perceived (pre-program) or actual barriers (post-program) to changing their child’s and family’s health. A further 3 items ask parents to report their confidence to 1) make healthy changes to child and family eating and activity patterns; 2) set limits regarding child food and eating; and 3) set limits regarding child activity/inactivity patterns. These questions are conceptually based on the Health Belief Model [46, 47].
			Attendance rates (P)	Program sign-in sheets	Purpose-developed sign in sheets for parents at each session
			Satisfaction with program and materials (P)	Purpose-developed questionnaire	Completed by parents at the end of program delivery. Includes satisfaction with program delivery and changes the family has made during the program.
ADOPTION	*Proportion of settings, practices, and plans that will adopt this intervention*	Organisation (Facilitator)	Number of facilitators trained (P)	Purpose-developed database and questionnaire	PEACH™ parent facilitator training logs
			Demographics (facilitators and services) (P)		Facilitator descriptors included gender, age, education, current employment status and experience in adult and child weight management in groups and 1:1
			Number of health services/other organisations engaged (P)		For purpose database containing details on each PEACH™ group including organisational setting
			Stakeholder interviews (P)	Purpose-developed interviews	Semi-structured interviews with facilitators, organisations and stakeholders
IMPLEMENTATION	*Extent to which the intervention is implemented as intended in the real world*	Organisation (Facilitator)	Number of facilitators who delivered groups and number of groups (P)	Purpose-developed database	For purpose database tracking facilitator involvement in the program (including demographics, training and program delivery)
			Adherence to program protocol and session outlines (fidelity) (P)	Purpose-developed questionnaire and session monitoring forms	Facilitators self-rate the quality of the group facilitation and content fidelity, for each session. It is based on a checklist developed for the NOURISH RCT [48].
MAINTENANCE	*Extent to which a program is sustained over time*	Organisation (Facilitator)	Workforce capacity change	This is beyond the scope of the PEACH™ delivery stage	To be determined
		Organisation (Health System)	Funding committed		
		Individual (Family)	Long term family impact		

^a^ *I* Impact evaluation*, O* Intervention outcomes*, P* Process evaluation

## Results

The evaluation framework of PEACH™ QLD was designed to meet the needs of the funder and as much as possible reflected its evolution from the RCT and community trial settings, comprising a range of outcome, impact and process evaluation indicators. It also included a range of broader environmental- and systems-based measures reflecting the Team’s experience in evaluating OPAL, South Australia’s settings-based community-wide childhood obesity prevention initiative [12]. Despite this broader evaluation design, there were some tensions between the evaluation framework and implementation/program delivery model which were not anticipated. This required a certain degree of reactivity to be introduced to the evaluation which in some cases presented significant practical/operational obstacles. For example, while community lifestyle programs need to be dynamic and have the flexibility to adapt to the needs of its participants during implementation, RCT intervention protocols are more strictly adhered to and do not normally change. In responding to participant needs and to improve participant engagement, there were iterative changes to program delivery including organisational settings, session scheduling, recruitment, and the order of session delivery. Some of these changes required modifications to the evaluation tools and consequent changes to the coding of variables collected and the syntax used for analyses. Furthermore, the governance under which the evaluation team operated required these changes to be submitted to multiple ethics committees for variations. Such changes were largely unanticipated, although in retrospect were necessary and appropriate actions to meet the needs of participants, service providers and the funding body. Achieving a balance between an evaluation framework that was flexible and adaptable enough to respond to change yet robust enough to maintain integrity was challenging. Additional learnings from key evaluation challenges encountered and actions undertaken during PEACH™ QLD are described in Table 3.

**Table 3:** Challenges arising from differences between RCT and implementation research paradigms

	Experience	Response	Key learnings
Ethics	*Ethics committees appeared to approach the Project from an RCT paradigm*		
	● Early requests from ethics committees included the addition of a control group and the de-identification of data prior to it being shared with the team. ● There was an apparent misunderstanding that ethics approval was required for the delivery of the program, as would be the case for an RCT, versus ethics approval for the collection of evaluation data, which is more appropriate for community program participants.	● Effort was made to develop relationships with ethics committees to enhance understanding of the Program and its implementation research approach.	● At ethics review, there is a need for the distinction between research-based practice and practise-based research such as program evaluation research.
Evaluation design	*Engagement challenges experienced during implementation required changes to inclusion criteria which are avoided in an RCT*		
	● In later stages of the program, inclusion criteria were expanded to include healthy weight children in addition to overweight and obese children in an effort to reduce the stigma of participation in a program for overweight/obese children.	● Questionnaires were updated to reflect the new criteria and changes in anthropometry needed to be reported separately for healthy weight children and the target population of children above a healthy weight. Data cleaning processes, data analysis syntax and feedback letters to families were tailored as needed.	● Make concessions for, and anticipate changes in, evaluation which are necessary when there are responsive changes in delivery of upscaled programs.
	*Evaluation length and consent process may have been unanticipated and burdensome on participants who signed up for a community program, and not an RCT*		
	● Participants enrolled in a community healthy lifestyle program and may not have considered themselves enrolled in a research project (c.f. RCT participants). Correspondingly, the lengthy participant information sheet and associated consent form required by ethics may have impacted participant engagement with evaluation and/or the program. ● In addition to consent procedures as required by ethics, evaluation questionnaires were lengthy	● An ethics modification was made in order to use data which were collected with implicit consent prior to and at sessions, without a signed consent form. ● The instrument for measuring physical activity was changed to a much shorter tool and onerous process evaluation items were omitted from questionnaires when further program changes were out of scope.	● The collection of some data for program monitoring without explicit participant consent (analogous to health service performance monitoring) should be considered reasonable and opt-out consent may be suitable for upscaled programs. ● Use a ‘minimalist or bare essentials’ lens when designing evaluation.
Data collection	*Research conducted in the world has a level of incomplete, unusable, and missing data, which is higher than research in a more tightly controlled RCT setting*		
	● Child facilitators conducted the anthropometric measures following training, using standardised equipment and protocols. These facilitators had various backgrounds (e.g. health professionals, teachers, team sport coaches) and some had limited experience in research and taking child measurements and may not have appreciated the implications for data collection. Consequently, there were some inaccuracies. ● Despite training and support on evaluation, parent facilitators may have held different perspectives on their role in the Program, particularly if they were operating in a service delivery paradigm rather than practice-based research. Hence, assisting with or ensuring data collection at sessions was not always seen as a priority – establishing rapport with parents was – and thus there was varied engagement with, and completion of evaluation.	● A height test to ensure correct assembly of the stadiometer improved the error rate and protocols for handling unreliable anthropometry data were established as part of quality assurance. ● All parent and child facilitators were trained, including the importance of data collection and evaluation processes, and the Evaluation Team monitored the return of evaluation data and sent reminders to facilitators to collect outstanding data at sessions or return outstanding questionnaires post-program.	● Where anthropometry is a key outcome, consider experienced or accredited personnel (e.g. International Society for the Advancement of Kinanthropometry) to take measures. ● Consider central and/or pre-program collection of evaluation data, to reduce burden on facilitators to collect data at early program sessions (especially for large groups).
	*Program re-enrolments violate rigourous RCT protocols but are optimal in an upscaled program*		
	● Families were able to enrol more than once which had a cascade effect as multiple ID numbers were given to the same child where their family re-enrolled. Multiple ID numbers were also administered when parents in a split family were enrolled in two separate groups, but the same child was participating in the program.	● Where a child had multiple enrolments and hence multiple study ID numbers, they had to be manually screened and excluded in data analysis so that each child was counted once.	● Flexibility to re-enrol in upscaled programs held in the community is desirable, however can lead to duplication of work: resources and time should be allocated to deal with data from these cases to manually exclude duplicates or reconcile sources of data when incomplete data are collected.
Process evaluation	*Process evaluation data are nice to have in an RCT but crucial for successful implementation of upscaled programs. Process data are challenging to identify and capture in real time*		
	● The Project Implementation Team desired ‘real-time’ feedback from programs to inform decision making during program implementation and were driven by meeting contracted enrolment targets. ● Rich program monitoring data were collected from a variety of sources during the project. These data were outside the formal evaluation framework and were not formally captured in databases in situ and analysed for reporting.	● The Evaluation Team was able to provide only limited process and outcome data in real-time outside the formal and contracted reporting schedule as data were collected only at program end and data cleaning/analysis processes were time-intensive. ● It took considerable time to reconcile data from multiple sources and prepare these data for analysis at the end of the project term.	● The identification, systematic capture, and analysis of process evaluation data from a range of sources may be better managed by the project delivery team who are in tune with program challenges and best equipped to respond to real-time feedback by making changes to delivery. ● Plan a priori for necessary expertise and budget to collect, manage, analyse and interpret process evaluation data in real time.

### Comprehensive evaluation is unlikely to be an expectation of participants in a community program

In large scale community trials, a trade-off exists between the evaluation data required by the funder and the actual or perceived response burden on participants. From PEACH™ RCT to community iterations of PEACH™, we simplified data collection in order to better meet participant expectations, minimise participant burden and to reduce evaluation costs (e.g. the omission of invasive blood sample collection and expensive blood biochemical analyses). PEACH™ QLD enrollees were participating in a healthy lifestyle program in the community setting and so were unlikely to consider themselves enrolled in a research project as participants in an RCT would. Furthermore, there was no requirement to provide data in order to participate and additionally no incentive to complete evaluation. Evaluation was hence simplified to collect only essential data in later iterations of the program, in order to minimise the influence of evaluation on program engagement and attendance. A specific example is the replacement of the lengthy CLASS questionnaire (Table 2) with the much shorter 6-item tool from the Youth Risk Behaviour Surveillance System questionnaire [13, 14]. It was our observation during the program that generally those who commenced questionnaires, including online, typically answered all or most questions, suggesting that evaluation length was not unacceptable.

### Duplication of data collection and complicated study ID numbers may unnecessarily increase burden on participants

Some data were collected as routine during the stages of program enquiry and enrolment for the purposes of determining eligibility and planning groups, including demographic information (child age, gender, residential postcode and parent-reported child height and weight). These data were also collected as part of the formal evaluation. The collection of data at multiple time points may have placed an unnecessary burden on participants. Additionally burdensome was the use of a 9-digit ID number conceived at the beginning of the Project to contain program delivery information including wave, site, group, facilitator, family and child information. Correct transcribing of the 9-digit ID number in evaluation by participants and facilitators was difficult and there were a number of errors. To maximise usable data, child date of birth was added to questionnaires to facilitate data matching and even this was inaccurately recorded a number of times. Future programs should consider simple ID numbers, with at least 2 other variables suitable to use as a cross check of information, such as date of birth and sex. Alternatively, data pertaining to program uptake and success (i.e. conversion from enquiry to enrolment, number of groups run, settings) may be better gathered by existing service provider information systems, where applicable, and linking evaluation data to a Medicare number or My Health Record as part of the National Digital Health Strategy. This would also enable ready access of the data to service providers and can be used internally to justify ongoing service delivery in their catchment, or for continuous quality improvement. Finally, we could have allocated participant ID numbers a posteriori, matching data to names rather than numbers, which may have placed greater focus on the Program delivery and less emphasis on the research component of the Project.

### Online data collection

Online data collection was a key strategy to maximise the amount of data collected, reduce facilitator burden, and enable ready access to evaluation data for monitoring and reporting. This was closely monitored and parent facilitators were informed of completion in real-time. Supplying an online link to the baseline questionnaire allowed parents to provide baseline data before their first session. This led to the collection of some baseline data – including parent and family socio-demographics – from 24% of enrolled families who did not attend any session. While these data were limited, they proved valuable in understanding the profile of families who enrolled but do not attend. Pre-program completion of questionnaires may have served as an early indication of engagement and commitment to the program.

Where baseline evaluation were not completed online before the sessions, trained facilitators played a supportive role in data collection at sessions as the geographical spread of groups precluded centralisation of data collection. This is in contrast to clinical research settings, where data collection during sessions/after program commencement is not appropriate as this can either distract from the delivery of the intervention or not serve as a true baseline measurement. Tablets provided for data collection at sessions were inefficient as devices had to be shared between group members and many participants were unfamiliar with their use. However, given the service delivery nature of the program, an additional session pre-program for data collection may not have been acceptable for families, and would have been a significant additional cost for the Project. Future programs need to develop their own strategies to optimise (and prioritise where necessary) the collection of data during program delivery.

### Missing data are to be expected in real world implementation research

Evaluation data, particularly at the child level, were largely uncollectable for those families who did not attend any program session (24%). Despite this non-attendance rate, parental response rate to questionnaires was reasonable (75%) and there was a modest level of questionnaire completeness (74%) leading to varying levels of missing outcome data. Of those children enrolled, 41% did not have any paired outcome data, largely due to non-attendance, at the final session in particular. Data collection was the responsibility of session facilitators (including health service clinicians and casually contracted professionals) and may not have been existing routine practice. To ensure more complete data collection in future, the importance of this task should be emphasised and supported so as to inform future practice and meet the ethical obligations of sharing and disseminating findings via peer-reviewed publications.

In addition, not all anticipated evaluation (Table 2) was able to be undertaken within the Project lifecycle. We had planned for stakeholder interviews in the evaluation framework under REAIM’s A: Adoption domain to investigate setting-specific barriers and enablers to program adoption within the health sector. However, during delivery of PEACH™ QLD there were changes in government policy leading to changes to the public health nutrition workforce [15]. We considered it untimely to conduct interviews on barriers and enablers of the project during this period of health care system flux and ensuing job uncertainty. Over the course of the project term, staff who delivered the Program were increasingly casual employees of QUT, operating outside the health sector. The timing and prioritisation of evaluation data collection (c.f. program implementation which is the priority) within a health care system should be carefully and cautiously appraised when systems are vulnerable and undergoing change.

## Discussion

While external evaluation has several strengths for research rigour and impartial expert assessment, it presented some challenges in the present study, particularly as the implementation and evaluation teams were two organisationally and geographically separate teams based in Brisbane, Queensland and Adelaide, South Australia, respectively. The main drivers for the Brisbane-based Project Implementation Team were ensuring a smooth running program and satisfying the tender milestone requirements, principally child enrolment numbers which were tied to financial payments. In contrast, the main driver for the Evaluation Team was the amount and quality of data collected with little direct connection to program facilitators.

Overall, attendance was a concern and the catalyst for modifications to program delivery, in particular a change from fortnightly, to weekly delivery of sessions 1–9. It was a challenge for the Evaluation Team to provide process evaluation data, in particular participant concerns, program satisfaction and acceptability in real-time to support this change as these data are only captured at the end of the 6-month program in the follow-up questionnaire. In response to this, additional qualitative research efforts to explore factors associated with enrolment [16] and engagement to better understand program access, need and attendance were driven by the Project Implementation Team. While not considered formal evaluation data, other information captured by the Project Implementation Team during administrative phone calls at enquiry and drop out times provided valuable insights into engagement with the program. Future programs should recognise and establish mechanisms for systematically capturing these data which informed program changes and sustainability of the program. Both the Project Implementation Team and the Evaluation teams worked hard to maintain a cohesive approach by having regular meetings and communicating well. Nevertheless, our experience from PEACH™ QLD has led us to question whether evaluation is best performed by two groups: process evaluation by the Project Implementation Team and outcome evaluation by an external evaluator. Implementation staff require data in a timely manner to facilitate program service delivery, however the present study consisted only of pre-post evaluation and the evaluation framework did not permit data collection or reporting in real time. During planning, teams need to work together to ensure clinical, service and system factors are adequately captured by the evaluation framework. During delivery, implementation staff must review process data sources and collection tools to ensure sufficient data are captured in order to address delivery challenges.

Implementation research is a relatively new and rapidly emerging field and theoretical approaches and frameworks underpinning implementation science have become more common over the past decade. There are now a number of conceptual frameworks for implementation including REAIM [11], Proctor [17, 18] and Consolidated Framework for Implementation Research [19]. While there is overlap between these frameworks, some elements or domains may be of greater importance in one implementation project compared to another. In practice, these models can be tailored to suit a specific implementation research program. In the PEACH™ QLD project, we largely carried forward the evaluation framework from the RCT and retrofitted this to the REAIM framework which we felt to be the best fit in 2012. While the Proctor and CFIR models were both published in 2009, there was at that time little exemplary literature regarding their use. On reflection, choosing the reporting framework a priori may have prompted us to capture additional data e.g. formal data on those who enquired about the program but did not enrol or attend, in order to describe ‘Reach’ in terms of those who do not participate as well as those who do. Ultimately, the scope of evaluation and project deliverables were negotiated with the funder, and the budget for evaluation was based on these deliverables. The funder of the program hence approved the evaluation framework based on the measurement and reporting of program outcomes to demonstrate effectiveness without concern for selection of a conceptual framework in which measures of effectiveness were embedded.

## Conclusion

In order to present a case for a program to be implemented long-term or become adopted as part of routine practice, it is critical that there is solid evidence, which requires collection of comprehensive and valid data. Collection of these data requires adequate funding for health service surveillance or specific program-targeted funding, as appropriate. Evaluation of upscaled programs in the real world is challenging as unlike gold standard efficacy studies in clinical or research environments, behavioural interventions and evaluation can evolve over time to meet participant need and fit within changing public health systems and settings. There may be some loss of control when upscaling potentially affecting participant engagement, intervention fidelity and hence evaluation rigour. Ultimately, there can be many trade-offs in translation research and researchers who find themselves working in this space need to be prepared to settle at times for something less than ideal.

Understanding practicalities in data collection and management may help others to design their data collection tools and processes to optimise data quality and ease and efficiency of data collection. Our learnings from the evaluation of PEACH™ as a service delivery program in Queensland provide new knowledge which can assist to inform the evaluation of future evidence-based, family-focussed interventions for children or other upscaled programs.

## Abbreviations

CONSORT: Consolidated Standards of Reporting Trials
HELPP: Healthy Eating and Activity through Positive Parenting
HL: healthy lifestyle
HREC: Human Research Ethics Committee
P: parenting
PEACH™: Parenting, Eating and Activity for Child Health
QLD: Queensland
RCT: Randomised Controlled Trial
REAIM: Reach, Effectiveness, Adoption, Implementation, and Maintenance
